# Universal Free School Meals and School and Student Outcomes: A Systematic Review

**DOI:** 10.1001/jamanetworkopen.2024.24082

**Published:** 2024-08-09

**Authors:** Maureen K. Spill, Rupal Trivedi, Rachel C. Thoerig, Arin A. Balalian, Marlene B. Schwartz, Craig Gundersen, Angela Odoms-Young, Elizabeth F. Racine, Margaret J. Foster, Julie S. Davis, Amanda J. MacFarlane

**Affiliations:** 1 Texas A&M Agriculture, Food & Nutrition Evidence Center, Fort Worth; 2 Department of Human Development and Family Science, College of Liberal Arts and Science, University of Connecticut, Storrs; 3 Department of Economics, Hankamer School of Business, Baylor University, Waco, Texas; 4 Division of Nutritional Sciences, College of Human Ecology, Cornell University, Ithaca, New York; 5 Department of Nutrition, College of Agriculture and Life Sciences, Texas A&M University, College Station; 6 Center for Systematic Reviews and Research Syntheses, University Libraries, Texas A&M University, College Station

## Abstract

**Importance:**

The White House National Strategy on Hunger, Nutrition, and Health included expanding free school meals to all students, regardless of income, which has sparked debate in the United States.

**Objective:**

To assess the association between universal free school meals (UFSMs) and school and student outcomes in the United States.

**Evidence Review:**

An expert panel–informed protocol was developed to evaluate intervention or cohort studies comparing UFSM programs, such as the Community Eligibility Provision (CEP), with non-UFSM programs in US schools from August 2012 (excluding 2020-2021 due to the COVID-19 pandemic) in peer-reviewed publications or government reports. Outcomes included meal participation rates, attendance, dietary intake and quality, food waste, economic impact, food insecurity, anthropometrics, disciplinary actions, stigma, and shaming. A search of Medline, Econlit, Business Source Ultimate, ERIC, Agricola, Cab Abstracts, and CINAHL was performed in April 2024. Two researchers screened articles for inclusion, extracted data, and assessed risk of bias, using the Risk of Bias in Nonrandomized Studies of Interventions tool, for each included study. Grading of Recommendations, Assessment, Development, and Evaluations was used to assess the certainty of evidence for each outcome.

**Findings:**

The search identified 2784 records, with 6 studies included, representing more than 11 000 elementary, middle, and high schools. Nonrandomized intervention studies performed difference-in-difference or rate ratio analysis to investigate CEP participation rates, attendance, anthropometrics, and/or suspensions. CEP was associated with increased lunch (3 studies; moderate certainty) and breakfast (1 study; very low certainty) participation. School attendance was unchanged or improved in schools with CEP compared with schools without UFSM (2 studies; low certainty). CEP was associated with lower obesity prevalence (1 study; very low certainty) and fewer suspensions (1 study; very low certainty). Reasons for downgrading the certainty ratings included indirectness (data not fully representative of the United States) and inconsistency (small number of studies limiting ability to assess consistency). Despite the limitations, the evidence reflected well-designed longitudinal intervention studies appropriate for decision-making.

**Conclusions and Relevance:**

In this systematic review, UFSMs were associated with increased meal participation, no or slight improvements in attendance, and decreased obesity prevalence and suspension rates; certainty of evidence was moderate for lunch participation and low or very low for other outcomes. Studies did not report several important outcomes, such as diet quality and food security, suggesting the need for more high-quality research encompassing policy-relevant indicators.

## Key Points

**Question:**

What is the association between universal free school meals (UFSMs) and school and student outcomes in US schools?

**Findings:**

In this systematic review of 6 studies comprising more than 11 000 schools, implementation of UFSM was associated with increased lunch (3 studies) and breakfast (1 study) participation, no change or modestly improved attendance (2 studies), and decreased obesity prevalence (1 study) and suspensions (1 study). The association with lunch participation had a moderate certainty of evidence, while the other associations had low or very low certainty of evidence.

**Meaning:**

In this study, implementation of UFSMs was associated with increased meal participation and potentially increased attendance and decreased obesity and suspensions.

## Teaser

This systematic review assesses whether expanding free school meals to all students is associated with improved participation in the program, attendance, student health, student behavior, and other student and school outcomes.

## Introduction

The White House National Strategy on Hunger, Nutrition, and Health was released in the fall of 2022 with the goal to “end hunger in America and increase healthy eating and physical activity by 2030 so fewer Americans experience diet-related diseases.”^1^ This strategy included “advancing a pathway to free school meals for all,” referred to as universal free school meals (UFSMs).^1^ Prior to allocating federal funds, research should be reviewed to assess the effectiveness of UFSMs.

Since 2014, the Community Eligibility Provision (CEP) has allowed federal reimbursement for qualifying schools^2^ to serve free meals to all students. Qualification into CEP is based on the percentage of the student body that qualifies for free meals using the traditional National School Lunch Program (NSLP) payment tiers.^3,4^ Students qualify for free meals if they come from households with an overall income of less than 130% of the federal poverty line or for reduced-price price meals if they come from households with an overall income between 130% and 185% of the federal poverty level.^5^ In the 2022 to 2023 school year, 82% of eligible schools had implemented CEP, providing 19.9 million children access to UFSMs.^6^ In the 2023 to 2024 school year, 9 states (California, Colorado, Maine, Massachusetts, Michigan, Minnesota, New Mexico, Nevada, and Vermont) went one step further by offering UFSMs to all schools regardless of CEP eligibility.^7^

There is debate in the United States on the expansion of UFSMs for all students regardless of income because of the additional spending that would be required. This debate should be informed by an up-to-date, high-quality systematic review (SR) that identifies and evaluates the strongest evidence available on the association between UFSMs and various school-level and student outcomes. In contrast to a previous SR,^21^ which incorporated cross-sectional and international evidence, the current SR assessed longitudinal studies measuring the associations of UFSMs in the United States with school- and student-level outcomes, including meal participation rates, attendance, dietary intake, diet quality, anthropometrics, economic impacts, disciplinary actions, food waste, stigma, and shaming.

## Methods

**Figure 1.**
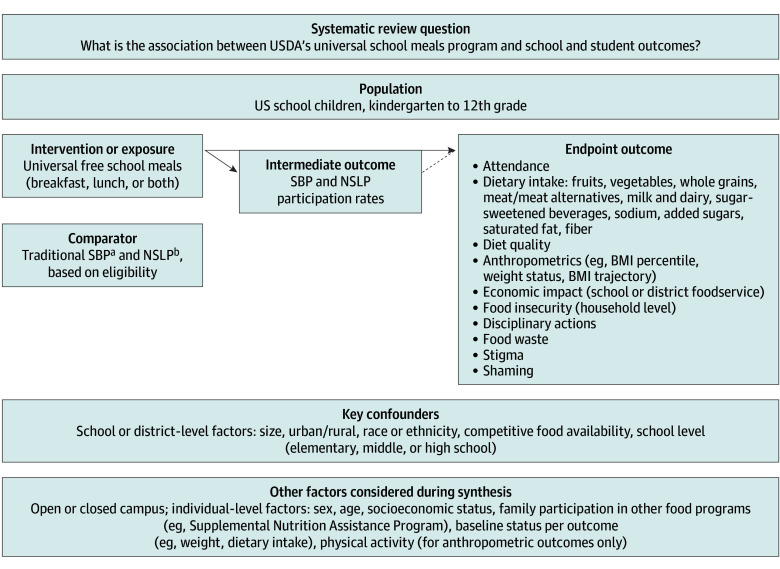
Analytic Framework BMI indicates body mass index; NSLP, National School Lunch Program; SBP, School Breakfast Program; and USDA, US Department of Agriculture.

A protocol was developed a priori, informed by subject matter experts (M.B.S., C.G., A.O.Y., and E.F.R.) with extensive knowledge and expertise in studying federal food assistance programs and registered in PROSPERO (CRD42023464854). The Population, Intervention, Comparator, Outcome (PICO) elements, key confounders, and other factors to be considered during synthesis are displayed in an analytic framework (Figure 1). An SR librarian (M.F.) ran an electronic search of peer-reviewed literature and government reports, 2 independent reviewers screened records, and 2 reviewers extracted and verified data. The risk of bias of each study was assessed by 2 independent reviewers using the study design–specific Cochrane Risk of Bias in Nonrandomized Studies of Interventions (ROBINS-I) tool, and the certainty of evidence rating was conducted using the Grading of Recommendations, Assessment, Development, and Evaluations (GRADE) approach. The eMethods, eTable 1, and eTable 2 in Supplement 1 provide details on the review methods, including search, eligibility criteria, screening, data extraction, risk of bias assessments, and certainty of evidence rating. This systematic review meets all Preferred Reporting Items for Systematic Reviews and Meta-analyses (PRISMA) reporting guideline criteria. DistillerSR was used to store and analyze data.

## Results

**Figure 2.**
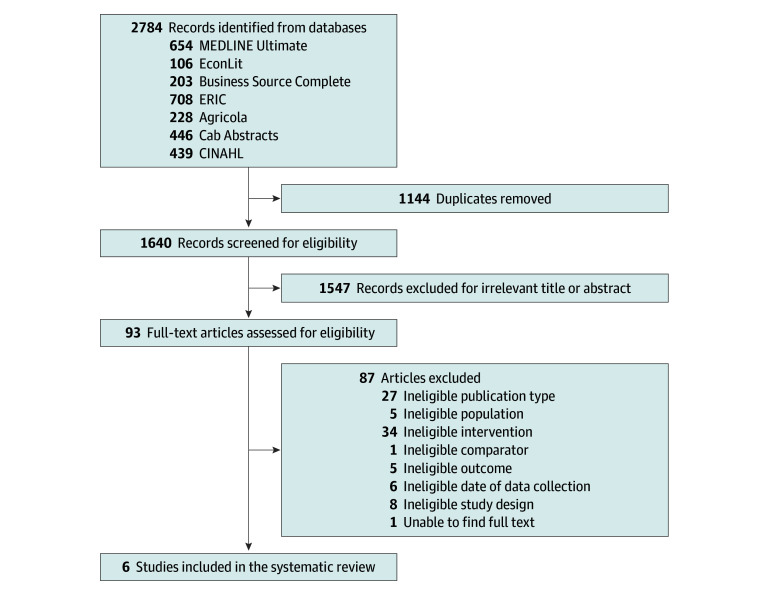
Flow Diagram of Screened and Selected Studies

The search returned 2784 records, of which 6 articles, representing more than 11 000 schools, were included in this SR^8,9,10,11,12,13^ (Figure 2). All included studies were nonrandomized intervention studies. The intervention in all studies was UFSMs via CEP; as such, schools in these studies that provided UFSMs had to apply and be eligible based on CEP requirements.^14^ The comparator group included schools not participating in CEP,^9,10^ CEP-eligible nonparticipating schools,^8^ or CEP-eligible schools that never participated.^11,12,13^ A difference-in-difference approach was applied in 5 studies to account for inherent differences between CEP participating and nonparticipating schools.^8,9,11,12,13^ The included studies reported 4 of the prioritized school and student outcomes: meal participation (breakfast and lunch), attendance rates, weight status (prevalence of obesity, overweight, and normal weight), and disciplinary actions (suspension rates). None of the eligible studies provided evidence on student dietary intake and diet quality, food waste, economic impact, household-level food insecurity, stigma, or shaming. The 6 included studies presented data from most schools within a state (California, Oregon, Pennsylvania, South Carolina, Texas, and Wisconsin). One article included data from Pennsylvania and Maryland, but only the data from Pennsylvania were included in this SR because the Maryland data were cross-sectional.^10^ The evaluated data ranged from the 2013 to 2014 to the 2018 to 2019 school years. All 6 studies assessed elementary school (ES) data, while 5 reported middle school (MS)^9,10,11,12,13^ and 4 reported high school (HS)^10,11,12,13^ data. All studies evaluated CEP interventions that provided lunch,^8,9,10,11,12,13^ and 3 also provided breakfast.^8,11,12^ The number of schools analyzed in each study ranged from 145 to 3531 schools. Five of the 6 studies described the racial distribution of students enrolled (Table 1).^8,9,11,12,13^

**Table 1.** Summary of Included Studies by Outcome^a^

Source	State	School years	School levels	Study groups	Sample size	Student race and ethnicity at baseline	Findings	Summary
**Lunch participation rates**								
Gordanier et al,^9^ 2019	South Carolina	2014-2015 to 2015-2016	ES and MS	Intervention: schools that implemented CEP in 2014-2015 or 2015-2016; comparator: any non-CEP participating schools	780 Total schools	CEP: approximately 28% White and 71% other race and ethnicity; non-CEP: approximately 61% White and 39% other race and ethnicity	All school levels: DID % (SE), 0.077 (0.013); *P* < .01 ES: DID % (SE), 0.082 (0.012); *P* < .01 MS: DID, % (SE), 0.063 (0.026); *P* < .05	There was a significant, positive association between CEP school lunch participation and the number of school lunches served for ESs and MSs in South Carolina.
Pokorney et al,^10^ 2019	Pennsylvania	2013-2014 to 2014-2015	ES, MS, and HS	Intervention: CEP schools; comparator: eligible, non-CEP schools	654 CEP schools and 1221 eligible, non-CEP schools	Not reported	All school levels: rate ratio, 1.08 (95% CI, 1.03-1.12) Students who would have qualified for free and reduced-price lunch at all school levels: rate ratio, 0.91 (95% CI, 0.86-0.96) Students who would not have qualified for free or reduced-price lunch at all school levels: rate ratio, 1.69 (95% CI, 1.11-2.56)	After CEP implementation, there was a significant increase in the rate of total lunches served in Pennsylvania.
Schneider et al,^11^ 2021	Texas	2013-2014 to 2018-2019	ES, MS, and HS	Intervention: CEP-eligible, ever-adopters; comparator: CEP-eligible, never-adopters	2797 CEP-eligible, ever-adopter schools and between 1237 and 2196 CEP-eligible never-adopter schools	CEP: approximately 15% White and 85% other race and ethnicity; non-CEP: 28% White and 82% other race and ethnicity	All school levels, excluding 3 summer mo: DID PP (SE), 4.61 (0.16); *P* < .001 All school levels, all 12 mo: DID PP (SE), 4.32 (0.15); *P* < .001	Compared with preintervention, there was a significant increase in lunch participation after CEP implementation in Texas.
**Breakfast participation rates**								
Schneider et al,^11^ 2021	Texas	2013-2014 to 2018-2019	ES, MS, and HS	Intervention: CEP-eligible, ever-adopters; comparator: CEP-eligible, never-adopters	2797 CEP-eligible, ever-adopter schools and between 1237 and 2196 CEP-eligible never-adopter schools	CEP: approximately 15% White and approximately 85% other race and ethnicity; non-CEP: 28% White and and 82% other race and ethnicity	All school levels excluding 3 summer mo: DID PP (SE), 4.64 (0.26); *P* < .001 All school levels, all 12 mo: DID PP (SE), 4.59 (0.26); *P* < .001	Compared with preintervention, there was a significant increase in breakfast participation after CEP implementation in Texas.
**Attendance**								
Bartfeld et al,^8^ 2019	Wisconsin	2013-2014 to 2015-2016	ES	Intervention: CEP schools; comparator: eligible, non-CEP schools	37 CEP schools and 108 eligible non-CEP schools	CEP schools, 58.4% of students belonged to a racial or ethnic minority group; eligible non-CEP schools, 36.2% of students belonged to a racial or ethnic minority group	ES attendance rates, 2014: coefficient (SE), 0.078 (0.187); *P* > .05 ES attendance rates, 2015: coefficient (SE), 0.320 (0.192); *P* > .05 ES low attendance (students attending fewer than 95% of available days), 2014: coefficient (SE), *−*0.015 (0.015); *P* > .05 ES low attendance, 2015: coefficient (SE), −0.035 (0.017); *P* < .05	There was not a significant change in attendance rates from baseline to the first or second year of CEP implementation; however, there was a significant improvement in percentage of students with low attendance from baseline to the first and second year of CEP implementation in Wisconsin.
Gordanier et al,^9^ 2019	South Carolina	2014-2015 to 2015-2016	ES and MS	Intervention: schools that implemented CEP in 2014-2015 or 2015-2016; comparator: any non-CEP participating schools	780 Total schools	CEP: approximately 28% White and 71% other race and ethnicity; non-CEP: 61% White and 39% other race and ethnicity	ES absenteeism: % (SE), −0.074 (0.138); *P* > .05 With time-varying controls: % (SE), −0.231 (0.111); *P* < .05 With inverse probability weighing: % (SE), −0.806 (0.152), *P* < .001 With propensity score trimming: % (SE), 0.212 (0.178); *P* > .10 MS absenteeism; % (SE): −0.520 (0.264); *P* < .05 With time-varying controls: % (SE), 0.421 (0.297); *P* > .10 With inverse probability weighing, % (SE), −0.353 (0.211); *P* < .10 With propensity score trimming: % (SE), −0.189 (0.352), *P* > .10	In South Carolina ESs and MSs, CEP-participating schools had better attendance rates (lower absenteeism) than non-CEP participating schools, although significance varied by analysis method.
**Anthropometrics**								
Localio et al,^12^ 2024	California	2013-2014 to 2018-2019	ES, MS, and HS	Intervention: CEP schools; comparator: eligible, non-CEP schools	1913 CEP schools and 1618 eligible, non-CEP schools	Overall approximately 11% White and 90% other race and ethnicity	Obesity prevalence, all school levels: All years: DID PP, −0.60 (95% CI, −1.07 to −0.14); *P* < .05 2014-2015: DID PP, −0.41 (95% CI, −1.60 to 0.78); *P* > .05 2015-2016: DID PP, −0.62 (95% CI, −1.55 to 0.30); *P* > .05 2016-2017: DID PP, −0.38 (95% CI, −1.20 to 0.43); *P* > .05 2017-2018: DID PP, −0.97 (95% CI, −2.16 to 0.21); *P* > .05 2018-2019: DID PP, −0.78 (95% CI, −1.39 to −0.16); *P* < .05 Overweight prevalence, all school levels: DID PP, 0.02 (95% CI, −0.38 to 0.42); *P* > .05 Normal weight prevalence: DID PP, 0.58 (95% CI, 0.08 to 1.08), *P* < .05	CEP implementation was associated with reduced obesity prevalence and increased prevalence of normal weight among students.
**Suspension rates**								
Domina et al,^13^ 2024	Oregon	2012-2013 to 2016-2017	ES, MS, and HS	Intervention: CEP schools, in 2014; comparator: eligible, non-CEP schools	300 CEP schools; 350 non-CEP schools	CEP schools in 2014, approximately 51% White and 49% other race and ethnicity; non-CEP schools in 2014, approximately 66% White and 34% other race and ethnicity	All school levels: DID coefficient (SE), −0.007 (0.003); *P* < .05 Kindergarten to grade 5; DID coefficient (SE), −0.004 (0.002); *P* > .05 Grades 6-8: DID coefficient (SE), −019 (0.007); *P* < .01 Grades 9-12: DID coefficient (SE), −0.025 (0.011); *P* < .05 ES panel: DID coefficient (SE), 0.001 (0.002); *P* > .05 Ninth grade panel: DID coefficient (SE), −0.029 (0.010); *P* < .01 Not low-income students: DID coefficient (SE), −0.002 (0.004); *P* > .05 Low-income students: DID coefficient (SE), −0.009 (0.003); *P* < .01 Students ineligible for school lunch: DID coefficient (SE), −0.002 (0.003); *P* > .05 Students eligible for reduced-price lunch: DID coefficient (SE), −0.007 (0.003); *P* < .05 Students eligible for free lunch, DID coefficient (SE), −0.011 (0.004); *P* < .01	CEP implementation was associated with reduced number of student suspensions.

Abbreviations: CEP, Community Eligibility Provision; DID, difference-in-difference analysis; ES, elementary school (generally grades kindergarten through 5); MS, middle school (generally grades 6 through 8); HS, high school (generally grades 9 through 12); PP, percentage point.

^a^ All included studies were nonrandomized studies of interventions.

Of the 3 studies that assessed an association between UFSMs and meal participation, evidence indicated that CEP interventions at lunch and breakfast were associated with increased meal participation (Table 1).^9,10,11^ Gordanier and colleagues^9^ assessed the association of CEP with the number of lunches served during the first and second year after intervention implementation. Results showed a significant, positive association between CEP and the number of lunches served in South Carolina. More specifically, there was a 7.7% increase in school meals overall (*P* < .01), which equated to 8.2% in ESs (*P* < .01) and 6.3% in MSs (*P* < .05).^9^ Pokorney et al,^10^ who compared the number of lunches served before and after CEP implementation, observed similar results. The study showed a significant 8% increase in the rate of lunches served after the CEP intervention among 1762 Pennsylvania ESs, MSs, and HSs (rate ratio, 1.08; 95% CI, 1.03-1.12).^10^ Additionally, this study found a 69% higher rate of meals served among students who would not have qualified for reduced-price meals in CEP schools compared with non-CEP schools (adjusted rate ratio, 1.69; 95% CI, 1.11-2.56).^10^ Schneider and colleagues^11^ examined participation rates before and after CEP implementation in Texas ESs, MSs, and HSs. After CEP implementation compared with before, there was a significant increase in lunch participation of 4.32 percentage points (*P* < .001) and breakfast participation of 4.59 percentage points (*P* < .001).^11^

Two studies assessed the association between UFSMs through CEP and attendance rates and found no change or an improvement in schools with CEP compared with schools without CEP (Table 1).^8,9^ Bartfeld et al^8^ evaluated differences in attendance rates (the percentage of school days attended) and low attendance (students attending fewer than 95% of available days) from baseline (2012 to 2013) to the first (2013 to 2014) and second (2014 to 2015) year of CEP implementation in Wisconsin ESs. There was a numerical increase in attendance rates (7.8 percentage point increase; *P* > .05) and decrease in low attendance (1.5 percentage point decrease; *P* > .05) between baseline (2012 to 2013) and the first year of implementation (2013 to 2014) for breakfast and lunch meals combined, but the results were not statistically significant.^8^ When comparing baseline (2012 to 2013) to the second year of CEP implementation (2014 to 2015), there was no significant difference in attendance rates (32.0 percentage point increase; *P* > .05); however, there was a small but significant decrease in low attendance rates (3.5 percentage point decrease; *P* < .05).^8^ Additionally, in South Carolina, CEP was associated with a significant decrease in absenteeism among MSs by approximately half (52%) of a day in the number of days a student is absent (*P* < .05), and a nonsignificant decrease in absenteeism among elementary schools.^9^

One study was identified that examined the association between UFSM and anthropometric measures.^12^ Localio et al^12^ used a difference-in-difference analysis to evaluate the association between implementation of CEP and the prevalence of obesity in California schools. CEP-participating schools had a 0.60 percentage point reduction in obesity prevalence (*P* < .05) and a 0.58 percentage point increase in prevalence of normal weight (*P* < .05) in comparison with CEP-eligible schools that never participated.^12^

One study assessed the associations between UFSM and disciplinary actions.^13^ For schools in Oregon, CEP implementation was associated with a reduced number of suspensions of 0.7 percentage points (*P* < .05).^13^ This was mainly driven by reduction in suspensions in MSs and HSs of 1.9 percentage points (*P* < .01) and 2.5 percent points (*P* < .05), respectively. These results remained significant for low-income students (−0.9 percentage points; *P* < .01), but not non–low-income students (−0.2 percentage points, *P* > .05). Additionally, considering lunch eligibility as a proxy for income, they found CEP was associated with reduced suspension rates for students that qualified for free (1.1 percentage points; *P* < .01) and reduced-price lunch (−0.7 percentage points; *P* < .05) and that there was a nonsignificant reduction for students ineligible for free or reduced-price lunch (−0.2 percentage points; *P* > .05).

Risk of bias was assessed for each study using the ROBINS-I tool, which is designed to identify sources of bias common to nonrandomized studies (Table 2). The study by Pokorney et al^10^ was rated as having a serious risk of bias^15^ because several key confounders, including school and district size, school level (ES, MS, and HS), urban and rural status, and student body race and ethnicity, were not accounted for in the design or analysis. The other 5 studies controlled for confounding through difference-in-difference analyses, thus also accounting for nonrandom selection bias, and were rated to have low risk of bias.^8,9,11^

**Table 2.** ROB Ratings for Nonrandomized Intervention Studies Using ROBINS-I Tool, by Outcome^a^

Source	Confounding	Selecting participants into the study	Classification of interventions	Deviations from intended interventions	Missing data	Measurement of the outcome	Selection of the reported results	Overall ROB rating
**Lunch participation rates**								
Gordanier et al,^9^ 2019	Low	Low	Low	Low	Low	Low	Low	Low
Pokorney et al,^10^ 2019^b^	Serious	Low	Low	Low	Low	Low	Low	Serious
Schneider et al,^11^ 2021	Low	Low	Low	Low	Low	Low	Low	Low
**Breakfast participation rates**								
Schneider et al,^11^ 2021	Low	Low	Low	Low	Low	Low	Low	Low
**Attendance**								
Bartfeld et al,^8^ 2019	Low	Low	Low	Low	Low	Low	Low	Low
Gordanier et al,^9^ 2019	Low	Low	Low	Low	Low	Low	Low	Low
**Anthropometrics**								
Localio et al,^12^ 2024	Low	Low	Low	Low	Low	Low	Low	Low
**Disciplinary actions**								
Domina et al,^13^ 2024	Low	Low	Low	Low	Low	Low	Low	Low

Abbreviations: ROB, risk of bias; ROBINS-I, Risk of Bias in Nonrandomized Studies of Interventions.

^a^ Rating options were low, moderate, serious, critical, no information.

^b^ The ROB assessment for Pokorney et al^10^ is based on Pennsylvania data only.

Using the GRADE approach, the certainty of evidence for the associations between UFSMs and meal participation was rated moderate and very low for lunch participation and breakfast participation, respectively. The certainty of the evidence was rated low for the associations between UFSM and attendance and very low for the associations between UFSM and anthropometrics and disciplinary actions (Table 3). The GRADE rating for risk of bias was informed by the risk-of-bias assessments conducted on each study using the ROBINS-I tool. Risk of bias was not downgraded because the risk-of-bias score was low for all or most studies informing each result (Table 2). Indirectness, which reflects the alignment between the PICO elements of the evidence to the predetermined PICO of the SR, was downgraded because the data were only from a few states and therefore may not be representative of the entire United States. There was only 1 study each for breakfast participation, anthropometrics, and disciplinary actions and only 2 studies for attendance, limiting the ability to assess inconsistency resulting in each being downgraded by 2 and 1 level, respectively. There were no downgrades or upgrades from the remaining GRADE domains (imprecision, publication bias, magnitude of effect, residual confounding, dose-response).

**Table 3.** GRADE Assessment for Certainty of Evidence by Outcome

Studies, No.	Design	Sources	Risk of bias^a^	Inconsistency^b^	Indirectness^b^	Imprecision^b^	Publication bias^c^	Magnitude of effect^d^	Influence of all plausible confounding^e^	Dose-response gradient^f^	Summary of findings	Certainty^g^
**Lunch participation rates**												
3	NRSI	Gordanier et al,^9^ 2019; Pokorney et al,^10^ 2019; Schneider et al,^11^ 2021	Not serious	Not serious	Serious; US population not fully represented as data are only from 3 states	Not serious	Not detected	No	No	No	Implementation of universal free meals at lunch was associated with increased lunch participation	Moderate
**Breakfast participation rates**												
1	NRSI	Schneider et al,^11^ 2021	Not serious	Very serious; only 1 study	Serious; US population not fully represented as data are only from 1 state	Not serious	Not detected	No	No	No	Implementation of universal free meals at breakfast was associated with increased breakfast participation	Very low
**Attendance**												
2	NRSI	Bartfeld et al,^8^ 2019; Gordanier et al,^9^ 2019	Not serious	Serious; results were consistent in direction, but only 2 studies	Serious; US population not fully represented as data are only from 2 states	Not serious	Not detected	No	No	No	Attendance rates did not change or were modestly improved in schools with universal free meals compared to schools without universal meals	Low
**Anthropometrics**												
1	NRSI	Localio et al,^12^ 2024	Not serious	Very serious; only 1 study	Serious; US population not fully represented as data are only from 1 state	Not serious	Not detected	No	No	No	Implementation of universal free school meals was associated with reduced prevalence of obese students, and increased prevalence of normal students	Very low
**Disciplinary Action**												
1	NRSI	Domina et al,^13^ 2024	Not serious	Very serious; only 1 study	Serious; US population not fully represented as data are only from 1 state	Not serious	Not detected	No	No	No	Implementation of universal free school meals was associated with reduced number of suspensions	Very low

Abbreviations: GRADE, Grading of Recommendations, Assessment, Development, and Evaluation; NRSI, nonrandomized study of interventions.

^a^ Downgrading domain. Response options: not serious, serious, very serious, or extremely serious.

^b^ Downgrading domain. Response options: not serious, serious, or very serious.

^c^ Downgrading domain. Response options: undetected or strongly detected.

^d^ Upgrading domain. Response options: no, large, or very large.

^e^ Upgrading domain. Response options: no, would reduce demonstrated effect, or would suggest spurious effect.

^f^ Upgrading domain. Response options: no or yes.

^g^ GRADE rating options: high, moderate, low, very low.

## Discussion

This SR found that the implementation of UFSMs was associated with increased meal participation rates among ES students and, to a lesser extent, MS students, with potential improvements for attendance. Furthermore, evidence suggested that UFSMs may be associated with lower obesity prevalence and disciplinary actions. While UFSMs ensure that students from lower-income households have access to healthy meals, regardless of whether they apply for free meals, it also benefits students from higher-income households.^16,17^ In fact, the greatest increase in school meal participation comes from students who would not have qualified for reduced-price or free meals through the traditional meal program.^10^ One study showed an increased rate (69%) of meals served among those who would not have qualified for reduced-price meals in CEP schools compared with non-CEP schools.^10^ This indicates that more students may consume meals at schools providing UFSM programs instead of bringing food from home or purchasing competitive foods at school from the à la carte line or vending machines, foods known to be less healthy.^18,19,20^ Furthermore, the consumption of school meals leads to increased fruits, vegetable, fish, whole grains, lean protein, and low-fat dairy intake, and decreased sugar, sodium, and fat intake, including saturated fat, particularly after the Healthy, Hunger Free Kids Act (HHFKA) implementation, which created stronger nutrition standards for foods sold in schools.^21,22,23,24^ Participation in school meals after implementation of HHFKA has been associated with lower incidence of obesity among participating students.^25,26^ Therefore, through increased meal participation, the UFSM programs may affect diet quality and health among students regardless of household income.

Increasing school meal participation rates may also benefit school and family finances. For example, when the New York City school district adopted UFSM through CEP in the 2017 to 2018 school year, it received approximately $61.8 million more in reimbursement that year compared with the previous year.^27^ Increased UFSM meal participation can improve financial burdens experienced and perceived by households and students. Marcus et al^28^ found that UFSMs through CEP reduced household food spending by approximately $11 per month, estimated to be 5% of monthly food purchases, and was associated with 5% fewer food insecure households nationwide.^28^

The association between UFSMs and meal participation is somewhat intuitive, but the impact of UFSMs on other outcomes is of great interest. Our results showed that attendance rates did not change or were modestly improved in schools with UFSMs compared with those without—consistent with previous reviews.^21^ Attendance may improve with UFSM programs, particularly for children from food-insecure households who have limited access to food outside of school and through reduced sickness-related absenteeism.^8,29,30^ However, many children from food-insecure households would have been eligible for free meals under the traditional school meal program. Therefore, the slight positive association between UFSMs and attendance is likely driven by students who would otherwise not qualify for free meals through the traditional school meal program.

Our review also identified evidence suggesting UFSMs may affect other school and student outcomes: weight status and disciplinary actions. One study found a significant reduction in the prevalence of obesity and an increased prevalence of normal weight among students in CEP schools.^12^ Another study found a reduction in student suspensions, mainly from MSs and HSs (grades 6-8 and 9-12),^13^ in schools that implemented CEP. These data are promising, but more research is needed to verify the impact of UFSM on these and other school and student outcomes.

The GRADE approach has 4 possible certainty-of-evidence ratings: high, moderate, low, and very low. Evidence supporting lunch participation findings was rated moderate. While the studies aligned with our PICO elements, the intent of this review was to inform US federal policy and therefore be generalizable to the US population. Because the population of the current evidence base was limited to 3 states, thereby potentially limiting its generalizability to all states, the rating was downgraded for indirectness. Evidence informing breakfast participation, attendance, weight status, and suspension rates were rated low or very low, largely due to indirectness and inconsistency because of the limited number of studies.

Supporting our findings on meal participation, a recent report by the Food Research & Action Center^31^ found that lunch participation increased from the prepandemic to the postpandemic periods when free meals were provided to all students in 5 states, and breakfast participation increased in 4 of the 5 states. This report used administrative data and data from annual surveys of state child nutrition officials in California, Maine, Massachusetts, Nevada, and Vermont, representing 5 states in addition to those captured in our review. These data suggest that our findings may indeed be generalizable to other states and therefore strengthen the finding that UFSM programs are associated with increased meal participation.

### Strengths and Limitations

The evidence included in the current review has several strengths. The nonrandomized intervention study design used in these studies provides longitudinal data on the impact of implementing UFSMs with comparator groups. Difference-in-difference analyses were conducted in 5 of the studies, reducing concern of nonrandom selection bias or bias from confounding. Sample sizes were large, covering most schools in 6 states. Data were collected from valid sources, namely state agencies, and when there was more than 1 study, the direction of effect was consistent.

Furthermore, this review was designed specifically to identify the strongest evidence to inform policy decisions. With this focus, inclusion was limited to studies in the United States with data starting from school year 2012 to 2013, when the HHFKA was implemented. Additionally, we excluded cross-sectional studies because they cannot infer causal relationships. Given the strict eligibility criteria, our review included fewer articles than other related reviews,^21,32^ but the included studies in those reviews were study designs with higher bias and lower certainty, such as cross-sectional studies. Here the included studies were all nonrandomized interventions of UFSMs that collected state-level school data over a minimum of a 1-year duration, allowing for an assessment of differences in outcomes over time in contrast to cross-sectional studies. We used rigorous review methods including an evaluation of the risk of bias using the Cochrane ROBINS-I tool designed specifically for nonrandomized studies of interventions. Furthermore, the GRADE approach was applied to provide certainty of evidence ratings––a critical piece for decision-makers. Considering the strengths and limitations of the primary evidence as well as the strict criteria and rigorous methods, this review provides meaningful evidence for policy makers and researchers.

This study also has limitations. The small number of included studies and limited number of important outcomes reported indicate a clear need for high-quality longitudinal research on the impact of UFSMs across all school and student outcomes. The 6 included studies provided evidence on the associations between UFSMs and participation rates, attendance, and anthropometric and disciplinary outcomes, but no included studies reported other outcomes prioritized by the subject matter experts, including dietary intake and quality, food waste, stigma, shaming, household level food insecurity, and the economic impact for schools. As of the 2023 to 2024 school year, CEP is available in all states, and 9 states offer UFSM programs regardless of eligibility into CEP.^7^ Data in additional states should be systematically collected and made accessible to researchers to evaluate the effectiveness of these UFSM programs. Furthermore, analyses addressing nonrandom selection bias, such as difference-in-difference approaches, should be applied to reduce the risk of bias and strengthen findings.

## Conclusions

In conclusion, this SR provides policymakers evaluating the impact of UFSMs with evidence that suggests that UFSMs are associated with increased meal participation and possibly improved attendance rates, reduced obesity prevalence, and reduced suspensions among US schools. These benefits could impact other outcomes, such as diet quality and academic outcomes, but more data are needed to confirm these hypotheses. Importantly, this review highlights the need for high-quality longitudinal evidence related to UFSMs across all outcomes.

## Notes

Supplement 1.
eMethods.
**eTable 1.** Database Search Strategy **eTable 2.** List of Studies Excluded at the Full Text Level, With Reason for Exclusion

Supplement 2.
Data Sharing Statement
