# Supplementary material for: Universal Free School Meals and School and Student Outcomes: A Systematic Review
Source: JAMA Netw Open. 2024 Aug 9;7(8):e2424082. doi: 10.1001/jamanetworkopen.2024.24082 (PMC11316229; doi:10.1001/jamanetworkopen.2024.24082)
Supplement: Supplement 1. — eMethods. eTable 1. Database Search Strategy eTable 2. List of Studies Excluded at the Full Text Level, With Reason for Exclusion [file jamanetwopen-e2424082-s001.pdf]

Supplemental Online Content

Spill MK, Trivedi R, Thoerig RC, et al. Universal free school meals and school and student outcomes. *JAMA Netw Open.* 2024;7(8):e2424082. doi:10.1001/jamanetworkopen.2024.24082

eMethods.

eTable 1. Database Search Strategy

eTable 2. List of Studies Excluded at the Full Text Level, With Reason for Exclusion

This supplemental material has been provided by the authors to give readers additional information about their work.

© 2024 Spill MK et al. *JAMA Network Open.*

eMethods

A. Protocol Development

The protocol was informed by subject matter experts (MBS, CG, AOY, EFR) with extensive knowledge and expertise in studying federal food assistance programs. The experts carefully refined the SR's PICO elements, determined eligibility criteria, assessed exposure and outcome ascertainment methods, and defined the key confounders that should be considered when assessing ROB. Their work was completed *a priori* to initiating the review. The protocol was registered in PROSPERO (CRD42023464854; https://www.crd.york.ac.uk/prospero/display_record.php?RecordID=464854).

B. PICO Framework and Eligibility Criteria

The Population, Intervention, Comparator, and Outcome (PICO) framework was used to inform the research question and the eligibility criteria for this SR (Figure 1).[1] To be included, study settings were US elementary, middle, or high schools during the academic school year, with UFSM as an intervention and the standard school meal program (with full price, free, and reduced price meals) as a comparator. The outcomes of interest included: SBP and/or NSLP participation rates, attendance rates, school meal dietary intake and quality, food waste, economic impact, household-level food insecurity, student-level anthropometrics, disciplinary actions, stigma, and shaming. Studies with data from the 2012/13 academic year (when compliance for updated nutritional standards was mandated by the USDA) and onward were included, except for March 2020 through August 2021 when schools were heavily disrupted due to COVID-19 mitigation policies.[2] Eligible study designs for the SR included randomized controlled trials, non-randomized studies of interventions (NRSIs) (including quasi-experimental and natural experiments), controlled before-and-after studies, and prospective cohort studies. Cross-sectional studies were excluded. Included articles were peer-reviewed publications or governmental reports. Articles that did not meet all the inclusion criteria were excluded.

C. Search and Screening Strategy

The search strategy was developed and performed by an experienced SR librarian (MF; supplementary table 1). Medical Subject Heading (MeSH) terms for universal free school meals, CEP, free and reduced-price meals, and breakfast and lunch meals were used to search the following databases to identify eligible articles: MEDLINE Ultimate, ERIC, Business Source Complete, EconLit, Agricola, Academic Search Ultimate, and CAB Abstracts databases. The search was last updated on April 2024.

The records were uploaded to DistillerSR, an online software used for literature management that enables reviewers to complete all stages of an SR independently. Two independent reviewers used the predetermined inclusion and exclusion criteria to first screen titles and abstracts, followed by full text screening of records included from the first-level screen. Any conflicting decisions were either resolved through a discussion between the two reviewers, or when appropriate, by another reviewer. Reasons for full text exclusions are provided in supplementary table 2.

D. Data Extraction

Study characteristics included study design, school level (elementary, middle, or high school), location, cohort name if applicable, school year(s), sample size, meal (breakfast and/or lunch), participation rates of the intervention, and the comparator involved in the study. The extracted participant data included sex, age, and race/ethnicity of the child, child's or parental socioeconomic status, and participation status for other US federal assistant programs (i.e. SNAP). Quantitative results between universal free school meals and outcomes of interests were extracted for each included article. Predetermined key confounding variables that studies did or did not consider were noted, in addition to other confounding factors assessed (figure 1). Reviewers noted limitations they identified and those listed in each study. For each included article, data were extracted by a reviewer, then the extracted data was verified for accuracy and completeness by a second reviewer. In the case of discrepancies between reviewers, conflicts were resolved through discussion or by input from a third reviewer.

E. Risk of Bias

The risk of bias (ROB) of each included article was assessed independently by two reviewers. To assess non-randomized studies of interventions (NRSI), Cochrane's Risk Of Bias In Non-randomized Studies - of Interventions (ROBINS-I) tool was used, which evaluates ROB for confounding, selection of participants, classification of interventions, deviation from the intended intervention, missing data, measurement of outcomes, and selection of the reported results.[3] The ROB for each domain was determined to be low, moderate, serious, critical, or no information.

© 2024 Spill MK et al. *JAMA Network Open.*

The overall study ROB rating was equivalent to the most severe ROB rating of any of the seven domain levels. Any disagreements in ROB rating at the domain and overall level were resolved between the two reviewers through discussion or if necessary, by another researcher.

F. Grading Certainty of Evidence

The Grading of Recommendations, Assessment, Development, and Evaluation (GRADE) approach was used to assess the certainty of evidence for each main finding. GRADE can be used for NRSIs, particularly when evidence from randomized studies is unavailable or not feasible.[4] GRADE provides a certainty of evidence rating by outcome and study design across the evidence.[4] For RCTs and NRSIs that are assessed using Cochrane ROB tools, the GRADE starts as high and is downgraded for every downgrade from the following domains: ROB, inconsistency, indirectness, imprecision, and publication bias. For NRSIs, ratings can be upgraded for each upgrade from: magnitude of effects, influence of all plausible residual confounding, and dose-response gradient. Overall, each outcome could be graded to have high, moderate, low, or very low level of certainty.[4] Two reviewers independently used GRADE to analyze the certainty of evidence, and consensus on decisions was reached through discussion or by the input of a third reviewer.

G. References

1.Riva JJ, Malik KM, Burnie SJ, Endicott AR, Busse JW. What is your research question? An introduction to the PICOT format for clinicians. J Can Chiropr Assoc. 2012;56(3):167-71.
2.Study NID. The impact of COVID-19 in education - more than a year of disruption. 2021.
3.Sterne JA, Hernán MA, Reeves BC, Savović J, Berkman ND, Viswanathan M, et al. ROBINS-I: a tool for assessing risk of bias in non-randomised studies of interventions. BMJ. 2016;355:i4919.
4.Schünemann H, Brożek J, Guyatt G, Oxman A. The GRADE handbook. Cochrane Collaboration London, UK; 2013.

© 2024 Spill MK et al. *JAMA Network Open.*

eTable 1. Database Search Strategy

Database	Date of search	Limit	Search 1 (S1)	Search 2 (S2)	Search 3 (S3)	Search 4 (S4)	Search 5 (S5)
Medline Ultimate (Ebsco)	29 April 2024	Limit 2012–current	TI((universal or free) n2 (meal or lunch)) OR AB ((universal or free) n2 (meal or lunch)) or TI ("National School Lunch" or "free or reduced-price meals") or AB ("National School Lunch" or "free or reduced-price meals") or TI ("Community Eligibility ") or AB ("Community Eligibility ")	(MH "Lunch" or MH "meals" or AB (meal* or lunch* or breakfast*) or TI (meal* or lunch* or breakfast*))	(MH "schools" or TI school* or AB school*)	S1 and S2 and S3	NA[a]
Econlit (Ebsco)	29 April 2024	Limit 2012–current	TI((universal or free) n2 (meal or lunch)) OR AB ((universal or free) n2 (meal or lunch)) or TI ("National School Lunch" or "free or reduced-price meals") or AB ("National School Lunch" or "free or reduced-price meals") or TI ("Community Eligibility ") or AB ("Community Eligibility ")	AB (meal* or lunch* or breakfast*) or TI (meal* or lunch* or breakfast*)	TI school* or AB school*	S1 and S2 and S3	NA
Business Source Ultimate (Ebsco)	29 April 2024	Limit 2012–current	DE "National school lunch program"	TI((universal or free) n2 (meal or lunch)) OR AB ((universal or free) n2 (meal or lunch)) or TI ("National School Lunch" or "free or reduced-price meals") or AB ("National School Lunch" or "free or reduced-price meals") or TI ("Community Eligibility ") or AB ("Community Eligibility ")	AB (meal* or lunch* or breakfast*) or TI (meal* or lunch* or breakfast*)	TI school* or AB school*	S1 or (S2 and S3 and S4)
**Database**	**Date of search**	**Limit**	**Search 1 (S1)**	**Search 2 (S2)**	**Search 3 (S3)**	**Search 4 (S4)**	**Search 5 (S5)**

© 2024 Spill MK et al. *JAMA Network Open.*

ERIC (Ebsco)	29 April 2024	Limit 2012–current	TI((universal or free) n2 (meal or lunch)) OR AB ((universal or free) n2 (meal or lunch)) or TI ("National School Lunch" or "free or reduced-price meals") or AB ("National School Lunch" or "free or reduced-price meals") or TI ("Community Eligibility ") or AB("Community Eligibility ")	DE "Lunch Programs"	(AB (meal* or lunch* or breakfast*) or TI (meal* or lunch* or breakfast*)) and (TI school* or AB school*)	S1 and (S2 or S3)	NA
Agricola (Ebsco)	29 April 2024	Limit 2012–current	TI((universal or free) n2 (meal or lunch)) OR AB ((universal or free) n2 (meal or lunch)) or TI ("National School Lunch" or "free or reduced-price meals") or AB ("National School Lunch" or "free or reduced-price meals") or TI ("Community Eligibility ") or AB("Community Eligibility ")	(AB (meal* or lunch* or breakfast*) or TI (meal* or lunch* or breakfast*)) and (TI school* or AB school*)	S1 and S2	NA	NA
Cab Abstracts (Ebsco)	29 April 2024	Limit 2012–current	TI((universal or free) n2 (meal or lunch)) OR AB ((universal or free) n2 (meal or lunch)) or TI ("National School Lunch" or "free or reduced-price meals") or AB ("National School Lunch" or "free or reduced-price meals") or TI ("Community Eligibility ") or AB("Community Eligibility ")	(DE "school meals" OR AB (meal* or lunch* or breakfast*) or TI (meal* or lunch* or breakfast*)) and (TI school* or AB school*)	S1 and S2	NA	NA
**Database**	**Date of search**	**Limit**	**Search 1 (S1)**	**Search 2 (S2)**	**Search 3 (S3)**	**Search 4 (S4)**	**Search 5 (S5)**
CINAHL (Ebsco)	29 April 2024	Limit 2012–current	TI((universal or free) n2 (meal or lunch)) OR AB ((universal or free) n2 (meal or lunch)) or TI ("National School Lunch" or "free or reduced-price meals") or AB ("National	(MH "Lunch") or AB (meal* or lunch* or breakfast*) or TI (meal* or lunch* or breakfast*)	(MH "Schools+") or TI school* or AB school*	S1 and S2 and S3	NA

© 2024 Spill MK et al. *JAMA Network Open.*

			School Lunch" or "free or reduced-price meals") or TI ("Community Eligibility ") or AB ("Community Eligibility ")				

© 2024 Spill MK et al. *JAMA Network Open.*

eTable 2. List of Studies Excluded at the Full Text Level, With Reason for Exclusion

Number	Source	Title	Reason for exclusion[a]
1.	Adams et al, (2022)	Nutrient Intake During School Lunch in Title I Elementary Schools With Universal Free Meals	Study design
2.	Alcaraz et al, (2014)	Cafeteria Staff Perceptions of the New USDA School Meal Standards	Intervention
3.	Andreyeva et al, (2021)	Universal School Meals in the US: What Can We Learn from the Community Eligibility Provision?	Date of data collection
4.	Argue et al, (2017)	Late Elementary Outcomes for Children Participating in Arkansas Better Chance (ABC) Program	Publication status
5.	Bartfeld et al, (2020)	The Community Eligibility Provision: Continuing the Century-Long Debate Over Universal Free School Meals	Publication status
6.	Bean et al, (2019)	A Cafeteria Personnel Intervention to Improve the School Food Environment	Intervention
7.	Bean et al, (2023)	Free Healthy School Meals for All as a Means to Advance Child Health Equity	Publication status
8.	Bergman et al, (2014)	School Lunch Before and After Implementation of the Healthy Hunger-Free Kids Act	Intervention
9.	Blagg et al, (2019)	How Restricting Categorical Eligibility for SNAP Affects Access to Free School Meals	Intervention
10.	Browne et al, (2017)	Summer Learning That Sticks	Intervention
11.	Bullock et al, (2022)	Associations Between a Universal Free Breakfast Policy and School Breakfast Program Participation, School Attendance, and Weight Status: A District-Wide Analysis	Date of data collection
12.	Burke et al, (2021)	A Randomized Controlled Trial of Three School Meals and Weekend Food Backpacks on Food Security in Virginia	Intervention
13.	Butcher et al, (2022)	Providing Free School Meals to Wealthy Students Does Not Help Children in Need	Publication status
14.	Campbell et al, (2021)	No Such Thing as a Free Lunch? Exploring The Consistency, Validity, and Uses of the 'Free School Meals' (FSM) Measure in the National Pupil Database	Population
15.	Carson et al, (2015)	Many Eligible Children Don't Participate in School Nutrition Programs: Reauthorization Offers Opportunities to Improve	Publication status

© 2024 Spill MK et al. *JAMA Network Open.*

16.	Center for Public Policy Priorities, (2012)	School Budget Cuts Threaten to Increase Summer Childhood Hunger	Publication status
17.	Cohen et al, (2022)	Implementation Of Universal School Meals During COVID-19 and Beyond: Challenges and Benefits for School Meals Programs in Maine	Date of data collection
18.	Cohen et al, (2023)	Universal Free School Meals: The Future of School Meal Programmes?	Publication status
19.	Colllier et al, (2015)	Free Lunch is a Good Thing for Children	Publication status
20.	Colorado Department of Education, (2013)	Colorado Growth Model--Brief Report: Student Growth Percentiles and FRL Status Accountability & Data Analysis Unit	Intervention
21.	Dalma et al, (2018)	Daily Distribution of Free Healthy School Meals or Food-Voucher Intervention? Perceptions and Attitudes of Parents and Educators	Population
22.	Demack et al, (2021)	Review of EEF Projects	Publication status
23.	Disiena et al, (2015)	Practice What You Preach: Does the National School Lunch Program Meet Nutritional Recommendations Set by Other USDA Programs?	Publication status
24.	Farris et al, (2014)	Elementary Parent Perceptions on Choosing to Participate in the National School Lunch Program or Packing Their Children's Lunches	Publication status
25.	Fazlul et al, (2023)	Free and Reduced-Price Meal Enrollment Does Not Measure Student Poverty: Evidence and Policy Significance	Intervention
26.	Ferris et al, (2022)	Increased School Breakfast Participation from Policy And Program Innovation: The Community Eligibility Provision and Breakfast After the Bell	Outcome
27.	FitzSimons et al, (2022)	Free School Meals for All is the Key to Supporting Education and Health Outcomes	Publication status
28.	Gearan et al, (2021)	Adolescent Participants in the School Lunch Program Consume More Nutritious Lunches but Their 24-Hour Diets Are Similar to Nonparticipants	Intervention
29.	Godfrey et al, (2013)	National School Lunch Nutrition Standards: Making Kids Hungry or Healthy?	Publication status
30.	Gordon et al, (2018)	School Nutrition and Student Discipline: Effects of Schoolwide Free Meals	Publication status

© 2024 Spill MK et al. *JAMA Network Open.*

31.	Gordon et al, (2021)	Schoolwide Free Meals and Student Discipline: Effects of the Community Eligibility Provision	Outcome
32.	Gothro et al, (2015)	The National School Lunch Program Direct Certification Improvement Study: Practices and Performance Report	Intervention
33.	Gross et al, (2021)	Household Food Security Status of Families With Children Attending Schools That Participate in the Community Eligibility Provision (CEP) and Those With Children Attending Schools That Are CEP-Eligible, but Not Participating	Study design
34.	Gutierrez et al, (2022)	User Guide to Model Estimates of Poverty in Schools	Intervention
35.	Handbury et al, (2021)	School Food Policy Affects Everyone: Retail Responses to the National School Lunch Program	Publication status
36.	Hauver et al, (2017)	Hunger in Our Midst: Civic Learning in the Context of Difficult Issues	Publication status
37.	Healthy Schools Network, Inc. (2016)	Towards Healthy Schools: Reducing Risks to Children	Intervention
38.	Hearst et al, (2019)	Breakfast is Brain Food? The Effect on Grade Point Average of a Rural Group Randomized Program to Promote School Breakfast	Outcome
39.	Hecht et al, (2021)	Universal Free School Meals Through the Community Eligibility Provision Maryland Food Service Provider Perspectives	Study design
40.	Henshaw, (2016)	School Leaders Renew Their Demands for Automatic Free School Meals Registration	Unable to find full text
41.	Henshaw et al, (2012)	Over Half of Children Living in Poverty Are Not Getting Free School Meals	Publication status
42.	Kannam et al, (2019)	Perceived Benefits and Barriers to Free Summer Meal Participation Among Parents in New York City	Population
43.	Kansas Association of School Boards (2016)	At-Risk Funding in Kansas: Free Lunch Status and At-Risk Status	Intervention
44.	Kirksey et al, (2021)	The Effect of Serving "Breakfast After-the-Bell" Meals on School Absenteeism: Comparing Results From Regression Discontinuity Designs	Intervention
45.	Kmietowicz et al, (2022)	Expand Free School Meals Urgently, Say Doctors and Campaigners	Publication status

© 2024 Spill MK et al. *JAMA Network Open.*

46.	Koedel et al, (2019)	Using Free Meal and Direct Certification Data to Proxy for Student Disadvantage in the Era of the Community Eligibility Provision	Publication status
47.	Koedel et al, (2020)	The Effect of the Community Eligibility Provision on the Ability of Free and Reduced-Price Meal Data to Identify Disadvantaged Students	Publication status
48.	Larin et al, (2023)	School Meal Programs: USDA Could Enhance Implementation of the Buy American Provision	Intervention
49.	Leos-Urbel et al, (2013)	Not Just for Poor Kids: The Impact of Universal Free School Breakfast on Meal Participation and Student Outcomes	Date of data collection
50.	Lindke et al, (2022)	Plate Waste Evaluation of Plant-Based Protein Entrees in National School Lunch Program	Intervention
51.	Litt et al, (2020)	Addressing Food Insecurity: An Evaluation of Factors Associated With Reach of a School-Based Summer Meals Program	Population
52.	Logan et al, (2014)	Community Eligibility Provision Evaluation	Study design
53.	Long et al, (2021)	Universal Free Meals Associated With Lower Meal Costs While Maintaining Nutritional Quality	Study design
54.	Marcus et al, (2022)	The Effect of Free School Meals on Household Food Purchases: Evidence From the Community Eligibility Provision	Date of data collection
55.	Meyer et al, (2021)	Lunch Skipping Behaviors Among Black And Hispanic Adolescents Who Receive Free School Meals	Intervention
56.	Moore et al, (2012)	Modeling of High Risk Indicators of Certification Error in the National School Lunch Program: Final Report	Intervention
57.	Moore et al, (2013)	Direct Certification in the National School Lunch Program: State Implementation Progress, School Year 2012-2013, Report to Congress	Intervention
58.	Moore et al, (2014)	The National School Lunch Program Direct Certification Improvement Study: Main Report	Intervention
59.	Moore et al, (2015)	Program Error in the National School Lunch Program and School Breakfast Program: Findings From the Second Access, Participation, Eligibility and Certification Study (APEC II)	Intervention
60.	Moore et al, (2015)	Direct Certification in the National School Lunch Program: State Implementation Progress, School Year 2013-2014	Intervention

© 2024 Spill MK et al. *JAMA Network Open.*

61.	Moore et al, (2016)	Direct Certification in the National School Lunch Program: State Implementation Progress, School Year 2014-2015. Report to Congress	Intervention
62.	Mosehauer et al, (2013)	The Future of School Breakfast: An Analysis of Evidence-Based Practices to Improve School Breakfast Participation in Washington State	Intervention
63.	Northey et al, (2024)	Data Analytics for Societal Challenges: Examining Student Participation in the National School Lunch Program	Publication status
64.	Poblacion et al, (2017)	Can Food Insecurity Be Reduced in the United States by Improving SNAP, WIC, and the Community Eligibility Provision?	Intervention
65.	Preston et al, (2013)	Assessment of the National School Lunch Program in a Subset of Schools in San Juan, Puerto Rico: Participants vs. Non-Participants	Intervention
66.	Ralston et al, (2015)	School Meals in Transition	Study design
67.	Ranalli et al, (2021)	Direct Certification in the National School Lunch Program State Implementation Progress Report to Congress, School Year 2017-2018 & School Year 2018-2019	Intervention
68.	Rogus et al, (2018)	Characteristics of School Districts Offering Free School Meals to All Students Through the Community Eligibility Provision of the National School Lunch Program	Outcome
69.	Rosen et al, (2022)	Ensuring Proper Benefits for Students in School Meal Programs	Intervention
70.	Rothbart et al, (2020)	Paying for Free Lunch: The Impact of CEP Universal Free Meals on Revenues, Spending, and Student Health	Publication status
71.	Ruffini et al, (2022)	Universal Access to Free School Meals and Student Achievement: Evidence From the Community Eligibility Provision	Study design
72.	Sather et al, (2021)	Impact of Summer Mobile Feeding Sites on Increasing Children's Access to Food	Intervention
73.	Schanzenbach et al, (2014)	Expanding the School Breakfast Program: Impacts on Children's Consumption, Nutrition and Health	Publication status
74.	Schermbeck et al, (2022)	Policies Shifting Towards Universal School Meals	Publication status
75.	Schirm et al, (2012)	Using American Community Survey Data to Expand Access to the School Meals Programs	Publication status

© 2024 Spill MK et al. *JAMA Network Open.*

76.	Schwartz et al, (2020)	Let Them Eat Lunch: The Impact of Universal Free Meals on Student Performance	Date of data collection
77.	Tan et al, (2020)	Community Eligibility Provision and School Meal Participation Among Student Subgroups	Study design
78.	Taylor et al, (2020)	Universal Free School Meal Programs in Vermont Show Multi-Domain Benefits	Comparator
79.	Trapp et al, (2018)	The Right to Taste: Conceptualizing the Nourishing Potential of School Lunch	Intervention
80.	Turner et al, (2019)	Community Eligibility and Other Provisions for Universal Free Meals at School: Impact on Student Breakfast and Lunch Participation in California Public Schools	Intervention
81.	USDA (2018)	Direct Certification in the National School Lunch Program: State Implementation Progress Report to Congress -- School Year 2015-2016 & School Year 2016-2017	Intervention
82.	Vericker et al, (2023)	USDA Summer Meals Program: Lack of Program Awareness Contributes to Unmet Need Among Nonparticipants	Intervention
83.	Walker et al, (2020)	Understanding the Challenges for Evidence-Informed School Improvement Support in Disadvantaged Schools: An Exploratory Study	Population
84.	Watkins et al, (2022)	Free School Meals to Alleviate Global Hunger	Publication status
85.	Watts et al, (2021)	The Campaign for Universal Free Lunch in New York City: Lessons Learned	Intervention
86.	Williams et al, (2022)	Impact of District-Wide Free Lunch on Third-Grade Students' Reading Comprehension	Outcome
87.	Yeado et al, (2014)	Public Schooling in Southeast Wisconsin: 2013-2014 [Summary]	Publication status

[a] Only one reason for exclusion required; therefore, there may be additional reasons for exclusion not listed.
