# Supplementary material for: Universal Free School Meals and School and Student Outcomes: A Systematic Review
Source: JAMA Netw Open. 2024 Aug 9;7(8):e2424082. doi: 10.1001/jamanetworkopen.2024.24082 (PMC11316229; doi:10.1001/jamanetworkopen.2024.24082)
Supplement: Supplement 2. — Data Sharing Statement [file jamanetwopen-e2424082-s002.pdf]

Data Sharing Statement

Spill. Universal Free School Meals and School and Student Outcomes. *JAMA Netw Open*. Published August 01, 2024. doi:10.1001/jamanetworkopen.2024.24082

Data
Data available: Yes
Data types: Other (please specify)
Additional Information: All included and excluded studies, search strategy will be made available.
How to access data: Excluded studies excluded at the level of full text screening are included in the supplement. Studies excluded at title and abstract screening will be made available by contacting the authors.
When available: With publication

Supporting Documents
Document types: None

Additional Information
Who can access the data: Anyone requesting the data.
Types of analyses: For any purpose
Mechanisms of data availability: With investigator support
